# Dabigatran-related serious medication errors: an analysis using data from VigiBase

**DOI:** 10.1007/s00228-024-03629-1

**Published:** 2024-01-29

**Authors:** Qingxia Zhang, Qian Ding, Qun-Ying Yue

**Affiliations:** 1 Department of Pharmacy, National Clinical Research Center for Geriatric Disease, Xuanwu Hospital Capital Medical University, Beijing, China; 2 Department of Pharmacy, National Center for Children’s Health, Beijing Children’s Hospital, Capital Medical University, Beijing, China; 3 Uppsala Monitoring Centre, Uppsala, Sweden

**Keywords:** Dabigatran, Medication error, Serious, Dosage error, Drug interaction, VigiBase

## Abstract

**Objective:**

To analyze the serious medication errors (MEs) on dabigatran, and their related factors, in order to avoid or reduce the occurrence of adverse events.

**Methods:**

Serious MEs related to dabigatran were extracted from the WHO global database of reported potential side effects of medicinal products (VigiBase) by using “Medication errors and other product use errors and issues” High Level Group Term (HLGT) of the international Medical Dictionary for Regulatory Activities (MedDRA). Well-documented reports, vigiGrade completeness score ≥ 0.80, or with an informative narrative were analyzed with a focus on the clinical features of the cases. The PCNE Classification for drug-related problems (DRP) was used to classify medication errors in our analysis of cases.

**Results:**

Until January 26, 2020, there were 453 cases with serious MEs related to dabigatran in VigiBase, and 113 were well-documented. Among these, 69 patients (61%) were hospitalized or had prolonged hospitalization, 16 (14%) had life-threatening events, and 12 (11%) died. The MEs occurred in the prescription phase in 77 cases, in administration in 35, and at the dispensing stage in one case. The MEs in prescription were related to a drug selection error in 44 cases (24 concerning contraindications and 20 drug interactions) and to dose error in 33 cases (17 with excessive dose; eight with insufficient frequency; four had an incorrect time; in three, the dose was too low; and in one, too frequent). The MEs in administration were medical-staff-related errors in five cases (three with wrong administration route, one administration omission, and one overdose), patient-related errors in 28 (14 insufficient dose or no administration, seven improper drug storage, four wrong administration method, and three over prescribed dose), and other errors in two (without efficacy monitoring). The dispensing error of a wrong drug strength occurred in a pharmacy. The main adverse events in the 113 patients were haemorrhage in 57 cases (50%) and ischemia in 29 cases (26%).

**Conclusion:**

Based on the analysis of reports in VigiBase, serious MEs related to dabigatran mainly occurred during prescription and administration. Although the incidence of MEs with clinical consequences in the use of dabigatran cannot be determined, attention should be paid to selection of the appropriate dose to a right patient in the prescription, and to patient compliance and storage in drug administration. The patient harm mainly manifested itself as bleeding or ischemia including fatal outcome in rare patients.

Oral anticoagulants are an important means to treat and prevent venous thromboembolism (VTE) and also play an important role in the prevention of stroke in patients with atrial fibrillation (AF) [1]. The traditional vitamin K antagonist, represented by warfarin, has a definite anticoagulant effect and low cost, but warfarin has complex pharmacokinetics, slow onset, and complex drug/food interactions [2]. Compared to warfarin, direct oral anticoagulants (DOACs) have faster onset, fewer drug/food interactions, predictable pharmacokinetics, and generally do not need frequent monitoring [3]. DOACs are safer than warfarin in stroke prevention in patients with non-valvular AF and also safer than heparin in the treatment and prevention of VTE, which has been proved in randomized clinical trials [4–9] and confirmed by global observational studies [10, 11]. At present, both VTE [12] and non-valvular AF [13] guidelines recommend DOACs as the first choice. Dabigatran (direct thrombin inhibitor) is one of the commonly used DOACs approved by the US FDA for stroke prevention in patients with non-valvular AF and VTE treatment and prevention.

The Institute for Safe Medicine Practices (ISMP) lists anticoagulants, including DOACs, as high alert drugs, because when they are wrongly used, the risk of serious injury to patients is high [14]. The American Geriatrics Society [15] listed dabigatran as a drug to be used with caution for the elderly, because it increases the risk of gastrointestinal bleeding in elderly people compared with warfarin. The analysis of the FDA Adverse Event Reporting System in 2016 [16] by ISMP found that anticoagulants caused 21,996 serious injury reports in the USA. The research of Piazza et al. [17] showed that adverse events caused by anticoagulants increased the mortality of patients within 30 days by 11%, of which nearly half (49%) was due to medication errors (MEs), the definition of which is “any preventable events that may cause or lead to inappropriate medication use or patient harm while the medication is in the control of the health care professional, patient or consumer” [18].

Our team’s previous research also found that 45% (15/33) of the fatal adverse events of dabigatran were related to MEs [19]. In order to understand the potential risks for the serious MEs reports related to dabigatran, we undertook this study to analyze the clinical characteristics of these cases from the WHO global database of reported potential side effects of medicinal products (VigiBase).

## Materials and methods

### Data source

The data of this study were derived from VigiBase until January 26, 2020, using the active ingredient dabigatran reported as suspected/interacting. All reports with serious “Medication errors and other product use errors and issues” (High Level Group Term (HLGT) of Medical Dictionary for Regulatory Activities (MedDRA) [20] were extracted.

### Data extraction

All the useful variables contained in the reports were considered, including the following: (1) case information: the category of reporters, the country and reporting date of cases; (2) patient information: age, gender, weight, relevant medical history; (3) medication information: suspected and concomitant medication indications, dosage regimen, and route of administration; (4) description of ME: error content, clinical manifestations, and results. The criterion for well-documented cases [21] was a vigiGrade completeness score ≥ 0.80, or the presence of an informative narrative (such as allowing causality assessment and analysis of their clinical characteristics). We used drug-related problems (DRP) [22] to classify medication errors in our analysis of cases.

MEs were reviewed and determined according to the product information by a graduate student in clinical pharmacy and a clinical pharmacist in cardiology department [23] and relevant diagnosis and treatment guidelines [12, 13] of dabigatran. At least one of the following criteria for serious adverse drug reactions were to be met [24]: death; life-threatening; carcinogenic, teratogenic, and birth defects; disabling that causes significant or permanent human disability or organ function damage; caused/prolonged hospitalization; and other medically important conditions, such as other significant medical events that may occur without treatment.

## Results

Until January 26, 2020, the database received 453 serious ME reports related to dabigatran from 33 countries. The highest proportion of reporter type was physicians (in 200 cases, 44%), followed by consumers or non-health professionals (96, 21%), pharmacists (95, 21%), and other health professionals (68, 15%) with the remainder unknown (6, 1.3%). Of the 427 patients with information on sex, 206 were male (48%), and 221 were female (52%). Information on patient age was available in 326 cases, and the median was 77 (range 2 ~ 97) years, of which 187 (57%) were 75 years or older.

A total of 113 of the 453 reports (25%) were classified as well-documented. The highest proportion of these were from physicians (74 cases, 65%), followed by pharmacists (20, 18%), consumers or non-health professionals (10, 8.9%), and other medical professionals (8, 7.1%), with one unknown reporter type (0.88%). Males accounted for 52% and female 48%, among the 107 patients with known sex. Information on patient age was given in 91 reports; the median was 76 (range 2–96) years, and 50 (55%) were 75 years or older. The events caused or prolonged hospitalization in 69 cases (61%), 16 (14%) being life-threatening, 15 (13%) noting other medically important conditions, 12 (11%) giving death, and one (0.88%) of disabling that caused significant or permanent human disability or organ function damage. The outcome was explicitly mentioned in 70 cases, of which 40 patients recovered (57%) and 30 had not recovered (43%) at the time of reporting. Among the 113 ME reports, 77 occurred during the prescription process, 35 at the administration stage, and one was in the dispensing. For details, see Table 1.

**Table 1:** Dabigatran-related serious medication errors

**ME process(n)**		**ME content (*n*)**			**Clinical manifestation**^c^**(*n*)**
		**Error**		**Correct**	
Prescription (77)	Drug selection (44)	Contraindications (24)	Severe renal insufficiency (7), mechanical valve (3), recent bleeding (2), combination of anticoagulant (warfarin (8), rivaroxaban (2), dabigatran (1) and fondaparinux (1))	Contraindication	Haemorrhage (19), ischemia (3)
		Interactions (20)	Pharmacodynamic interaction (aspirin (7), aspirin + clopidogrel (2), aspirin + ticagrelor (1), aspirin + citalopram(1), steroid (1), clopidogrel (1), diclofenac (1), paracetamol 4 g/day(1)) Pharmacokinetic interaction (amiodarone (3), verapamil (2))	Consider therapy modification	Haemorrhage (16), prolonged coagulation time (1), ischemia (1), cerebrovascular accident (1)
	Dose selection (33)	Excessive dose (14)	AF: 300 mg (6), patients over 80 years old: 150 mg (4), patients over 80 years old with renal insufficiency: 150 mg (1), patients with renal insufficiency: 150 mg (1), multiple risk factors^a^ for increased bleeding: 110 mg, bid (1), primary prevention of orthopaedic surgery in patients over 75 years old: 220 mg (1)	Dosage of each time 150 mg: AF/primary prevention of orthopaedic surgery in patients over 75 years old 110 mg: patients over 80 years old/patients with renal insufficiency 75 mg: multiple risk factors^a^ for increased bleeding	Haemorrhage (11), acute renal insufficiency (1)
		Insufficient frequency	Qd (8) [AF (7), VTE (1)]	AF/VTE: bid	Ischemic stroke (5), cerebrovascular accident (1), DVT (1), thrombophlebitis (1)
		Incorrect duration (4)	Anticoagulants during perioperative period: premature withdrawal (2), non-withdrawal (2)		Cerebrovascular accident (2) and haemorrhage (2)
		Low dose (3)	Severe obesity^b^(153 kg, BMI 44.7 kg/m2): 150 mg bid (1), 110 mg in the morning and 150 mg in the evening for AF patient (1), 150 mg but wrongly prescribed 110 mg for AF patient (1)	AF: 150 mg each time	Ischemic stroke (3)
		Excessive dose and insufficient frequency (3)	AF: 220 mg qd (3)	AF: 150 mg bid	Haemorrhage(3)
		Excessive frequency (1)	THR surgery: 75 mg bid	THR surgery: qd	Haemorrhage
Dispensing (1)	Preparing (1)	Wrong drug strength (1)	Dispensed mistakenly 110 mg/capsule	150 mg/capsule	Ischemic stroke
Administration (35)	Medical staff related (5)	Wrong administration route (3)	Gastric tube (2), intraocular (1)	Do not open or break	Blindness (1)
		Administration omission (1)	The patient thought that it was other drugs that were not taken because being not informed about the change of drug packaging	Remind patients of packaging changes	
		Overdose (1)	Two capsules (75 mg/capsule) bid, previously; drug strength changed to 150 mg/capsule, still take 2 capsules (300 mg) bid	1 capsule (150 mg/capsule) bid	Haemorrhage
	Patient related (28)	Insufficient dose or administration omission (14)	Drug omission (9), self-decrement (3), self-withdrawal (2)	Do not skip or stop the drug	Ischemic stroke (8), cerebrovascular accident (3), PE (1), DVT (1)
		Improper drug storage (7)	Capsules were stored outsides its original package (6), child ingestion by mistake (1)	Keep in original packaging; Keep away from children	Ischemic stroke (4), cerebrovascular accident (1)
		Wrong administration method (4)	Capsules were opened (4)	Do not open or break	Haemorrhage (1), ischemic stroke (1), cerebrovascular accident (1), epigastric pain (1)
		Over prescription dose (3)	Patients forgot to reduce the dosage (1), self-dosing (2)	Do not change the dose by oneself	Haemorrhage (1)
	Other (2)	Without efficacy monitoring (2)	Hypertension (1) and falls (1)	Control blood pressure and prevent falls	Haemorrhage (2)

*n *= 113

*AF* atrial fibrillation, *DVT* deep venous thrombosis, *ME* medication error, *PE* pulmonary embolism, *THR* total hip replacement, *VTE* venous thromboembolism, *qd* once a day, *bid* twice a day

^a^Factors include the elderly, renal insufficiency, low body weight, and hypertension

^b^Serum concentration of dabigatran below the therapeutic range led to treatment failure [29]

^c^Only those with clear clinical manifestations related to drugs and ME are listed, while those with unclear and unknown symptoms are not listed

## Discussion

The prescription MEs is mainly related to drug selection or dosage errors, with the former concerning principally contraindications and drug interactions.

The contraindications included combined use of anticoagulants, use in patients with severe renal insufficiency, mechanical valves, or recent bleeding history. The contraindications specified in the product information [23] of dabigatran include adult patients with severe renal function impairment (CrCL < 30 mL/min), artificial heart valves requiring anticoagulation treatment, patients with clinically significant bleeding, and use of any other anticoagulant drugs (except in the bridging period). Patients with recent bleeding or concomitant anticoagulants had a significantly increased risk of bleeding. As patients with severe renal insufficiency, or artificial heart valves, were excluded from the key randomized controlled trials, there is no evidence for the efficacy and safety of dabigatran in these groups [13]. However, recent registry study [25] has shown that DOAC use in patients with atrial fibrillation after transcatheter aortic valve replacement (TAVR) had lower bleeding risks with comparable effect (stroke risk) compared with vitamin K antagonists.

Drug interactions can be divided into pharmacodynamic or pharmacokinetic interactions. Antiplatelet drugs (e.g., aspirin, clopidogrel, and ticagrelor), citalopram, glucocorticoid, and diclofenac may increase the bleeding risk of patients. Excessive acetaminophen can lead to liver injury, while liver injury may lead to coagulation dysfunction [26]. The combination of these drugs with dabigatran will increase the risk of bleeding. Dabigatran is the substrate of the efflux transporter P-gp. The combination of dabigatran with the strong P-gp inhibitor amiodarone will increase the blood concentration of dabigatran by between 12 and 60% [13]. If verapamil is taken at the same time, the blood concentration of dabigatran may increase by between 12 and 180% [13].

Excessive use of DOACs leads to a higher risk of bleeding, while under dosing increases the risk of stroke and systemic embolism. The study by Shen et al. [27] showed that the prevalence rate of off-label DOACs use was estimated to be 24% in the AF population. However, Godino et al. [28] pointed out that although inappropriate dosage prescriptions for DOACs were frequent, the available data on the clinical effects of such inappropriate prescriptions are limited. In our study, cases with an insufficient dose were few, and the correlation between dose error and patient age and gender could not be obtained. Gozzo et al. [29] showed that dosage errors were related to older age, kidney disease, bleeding risk, and the simultaneous use of drugs that were linked to bleeding but were not related to CHA2DS2-VASc and HAS-BLED. Registry data showed that DOACs over- and underdosing are associated with increased risk for adverse events [30]. Therefore, we should be alert to the risks of excessive/insufficient dosage and pro-actively observe the bleeding/thrombosis risk factors of patients and adjust the dosage in a timely fashion.

Patients (28 cases, 80%) were mainly responsible for the administration MEs. The principal reason for patient-related ME was drug compliance, which resulted in ischemic events in patients due to drug omission or self-withdrawal. Because the action time of DOACs is short, the anticoagulation effect almost disappears between 12 and 24 h after drug withdrawal [31]. Therefore, it is necessary to strengthen patient education about DOACs, which can refer to the ISMP template [32].

Regarding patient-related MEs, attention should also be paid to the method of drug storage and administration. The dosage form of dabigatran is a capsule. The Summary of Product Characteristics (SmPC) [23] of dabigatran state that dabigatran should be stored in the original package to prevent moisture. Therefore, it should not be taken out and put in the kit and should not be opened or chewed. In this study, a child took dabigatran by mistake, leading to hospital observation. As a high alert drug, DOACs should be kept away from children, which was not mentioned in the product information. Dabigatran is a pro-drug, and the bioavailability increases by 75% if the capsule is opened [23]. Therefore, dabigatran should not be opened for administration, and the whole capsule swallowed.

In this study, there was one serious event caused by incorrect dispensing, suggesting that pharmacists should carefully check the information of each patient and drug when providing the high-alert drug DOACs.

Patient-related problems are the main mistakes found when taking medicines. Inadequate awareness in patients of the precautions needed when taking DOACs may lead to serious MEs. Therefore, attention should be paid to drug guidance for patients taking DOACs.

As with all spontaneous reporting databases, VigiBase has its limitations, starting with under-reporting and selective reporting. Secondly, due to the nature of spontaneous reporting, in most cases (about 70%), there is a lack of information on important clinical parameters and concomitant diseases or other factors. Therefore, we selected and analyzed the reports containing more complete information. In addition, because there is no denominator in VigiBase, it is impossible to estimate the risk incidence rate. We only qualitatively assessed the risk factors of serious adverse events in the study population based on the data of VigiBase, rather than quantitatively assessed them. In addition, we used MedDRA HLGT “Medical errors and other product use errors and issues” as the search strategy, which does include “Labelled drug-drug interaction issue (PT)” and “Labelled drug-drug interaction medication error (PT).” However, terms such as drug interaction (PT), drug-disease interaction (PT), drug-genetic interaction (PT), and food interaction (PT) that belong to another SOC “General disorders and administration site conditions” were not captured. Although our search strategy maintains a certain level of specificity for ME cases, it may omit some drug interactions which could be labelled but not reported as medication error, thereby leading to under-estimation of the number of cases. Finally, there were no comparators used in this study, and risks associated with MEs were not put into perspective of the benefit. Some recent registry findings, however, were commented above.

In conclusion, serious MEs including fatal cases related to dabigatran have been reported to VigiBase. Based on the analysis of these reports, the MEs mainly occurred in prescription and administration. Although the incidence of MEs with clinical consequences in the use of dabigatran cannot be determined, attention should be paid to selection of the appropriate dose to a right patient in the prescription and to patient compliance and storage in drug administration.

## Data Availability

The original contributions presented in the study are included in the article/Supplementary Material. Further inquiries can be directed to the corresponding authors.
